# Effects of two-stage preterm formulas on growth, nutritional biomarkers, and neurodevelopment in preterm infants

**DOI:** 10.3389/fped.2024.1427050

**Published:** 2024-11-22

**Authors:** Przemko Kwinta, Svilena Lazarova, Klaudia Demová, Yipu Chen, Mickaël Hartweg, Laura-Florina Krattinger, Cecilia Fumero, Aleksandra Buczyńska, Wojciech Durlak, Zuzana Uhrikova, Marek Kozar, Tinu Mary Samuel, Mirko Zibolen

**Affiliations:** ^1^ Department of Pediatrics, Jagiellonian University, Krakow, Poland; ^2^ Nestlé Product Technology Center—Nutrition, Vevey, Switzerland; ^3^ Department of Neonatology, Faculty Hospital Nové Zámky, Nové Zámky, Slovakia; ^4^ Clinical Research Unit, Nestlé Research, Lausanne, Switzerland; ^5^ Jessenius Faculty of Medicine, Martin/Comenius University, Bratislava, Slovakia

**Keywords:** growth, nutritional biomarkers, feeding tolerance, neurodevelopment, infant development, preterm infants, preterm formula

## Abstract

**Background:**

Formula-fed preterm infants require nutrient-enriched formulas with optimized protein levels to support growth and neurodevelopment. The purpose of this study was to evaluate the safety, tolerability, and effectiveness of a new liquid two-staged formula system designed to provide tailored nutrition during hospital stay and after discharge.

**Methods:**

Male and female very-low-birth-weight preterm infants (birth weight ≤1,500 g; gestational age ≤32 weeks) were recruited from three neonatal units in Poland and Slovakia in a prospective, open-label, interventional study. Stage 1 formula providing 3.6 g intact protein/100 kcal was consumed from enrollment until reaching 1,800 g, followed by a post-discharge (PD) Stage 2 formula with 2.8 g/100 kcal protein, which was consumed for 30 days. Weight gain velocity (WGV in g/kg/day) between the first day of achieving full enteral feeding (FEF D1 rate of 150 ml/kg/day and cessation of parenteral feeding) and day reaching 1,800 g was compared to the minimally required WGV (15 g/kg/day) for non-inferiority (primary endpoint), and to the Fenton median growth rate for superiority (17.3 g/kg/day), adjusting for sex, gestational age, site, visit, and WGV. Changes in *z*-scores, feeding tolerance, nutritional biomarker status, and safety were also assessed from FEF D1 to 30 days PD. In an observational follow-up at 2 years of age, neurodevelopment was evaluated using the Bayley Scales of Infant and Toddler Development (BSID-III).

**Results:**

Adjusted weight gain velocity (95% CI) between the first day of full enteral feeding and day reaching 1,800 g in per protocol (PP, *N* = 18) was 23.0 (20.1–25.9) g/kg/day; lower limit of the 95% CIs exceeded the non-inferiority margin (15 g/kg/day, *p* < 0.001) and the superiority margin (17.3 g/kg/day, *p* < 0.001). Mean stool frequency ranged from 2.5 to 3.3 stools per day. The two-stage formula supported adequate growth patterns throughout the study and nutritional biomarkers of protein and mineral status were within normal ranges. At 24 months corrected age, the mean ± SD of the BSID cognitive scale was 97.3 ± 13.9 in PP, with all infants achieving a score >70. None of the adverse events reported were related to the study formulas.

**Conclusion:**

The two-stage preterm formulas supported postnatal weight gain, adequate growth, cognitive development within normal ranges, and a safe profile of protein and bone biomarkers.

**Clinical Trial Registration:**

Clinicaltrials.gov registration, NCT03728764, NCT04962035.

## Introduction

Preterm infants (<37 weeks gestation), particularly very preterm infants (≥28 and <32 weeks gestation), are born with substantial nutrient deficits and are at risk of multiple growth and developmental delays (1–3), which can impact skeletal growth, bone mass accretion, cognition, and lead to compromised metabolic health and poor educational performance later in life (4). Breastfeeding is preferred in all infants for optimal quality of growth (5, 6). Nevertheless, when a mother's own milk is unavailable or insufficient, donor human milk or preterm formulas represent alternative nutritional sources (7).

To meet the increased nutritional needs of preterm infants, recent expert guidelines by the European Society for Pediatric Gastroenterology, Hepatology, and Nutrition Committee (ESPGHAN) in 2022 called for protein intakes of 3.5–4.0 (up to 4.5) g/kg body weight/day and higher total energy intake of ∼115–160 kcal/kg/day (8). Furthermore, a previous 2006 ESPGHAN position statement recommended that a special nutrient-enriched post-discharge (PD) formula, differing in composition in comparison to a neonatal formula, should be used to tackle the presence of cumulative nutritional deficits in formula-fed preterm infants post hospitalization (9). Although a growing number of studies have utilized various standard and energy/protein-enriched formulas to evaluate growth and neurodevelopment in preterm infants, findings for improved growth have been consistent only with higher protein and energy quantity in in-hospital postnatal formulas (10, 11). Research on growth with post-discharge formulas has been inconclusive (12, 13) and research on cognition has been insufficient to show a beneficial effect for any type of preterm formula (11–14). To our knowledge, no studies in preterm infants have so far explored the combined effect of a two-stage feeding system, designed in line with the most recent expert recommendations and addressing the different nutritional needs during hospitalization and post-discharge, on growth and neurodevelopment.

We aimed to assess the safety, tolerability, and effectiveness of a new protein-enriched two-stage liquid preterm feeding system designed to provide tailored nutrition during hospital stay and after discharge in very-low-birth-weight (VLBW) infants (birth weight ≤1,500 g). A prospective, open-label, single-arm interventional study was conducted with a primary objective of evaluating weight gain velocity (WGV in g/kg/day) from day 1 of full enteral feeding (FEF D1) to the day 1,800 g were reached, and secondary objectives of evaluating other growth parameters, feeding tolerance, time to full enteral feeding, biomarkers of nutritional status, and adverse events (AEs). As a follow-up to this interventional study, an observational study was conducted with a primary objective of characterizing cognitive outcomes at 24 months corrected age (CA) utilizing the *Bayley Scales of Infant and Toddler Development*—Third Edition (BSID-III) (15). Secondary objectives included the assessment of developmental milestones, temperament, healthcare usage and hospitalizations, growth, and feeding practice. We hypothesized that mean adjusted weight gain velocity between FEF D1 and day reaching 1,800 g will be non-inferior to the recommended intrauterine growth rate of 15 g/kg/day (16).

## Methods

### Study design and participants

This was a multicenter, prospective, open-label, single-arm interventional study (NCT03728764) in preterm infants younger than 10 days in the neonatal care unit (NICU) upon enrollment and with a follow-up time of 30 days after discharge. The study was conducted at three NICUs, including the Children's University Hospital (Poland), Faculty Hospital Nové Zámky (Slovakia), and Martin Comenius University Hospital (Slovakia), from October 2018 to December 2019. A single-arm study design was chosen due to a very small population pool of predominantly formula-fed preterm infants. Infants who participated in the feeding study were eligible for enrollment in a follow-up observational study with assessment visits at 18 and 24 months of CA. The studies complied with the Declaration of Helsinki and the International Conference on Harmonization Guidelines for Good Clinical Practice and were approved by each site's Independent Ethics Committee. Written informed consent was obtained from each infant's parent(s) or legal representative prior to study entry.

Male and female clinically stable, VLBW infants [27–32 weeks of gestational age (wGA); birth weight ≤1,500 g] without access to a sufficient supply of breast milk and able to start formula after 24 h of trophic feeding were enrolled within the first 10 days (240 h) after birth. Appropriate for gestational age was defined as birth weight ≥10th and ≤90th percentiles on the Fenton Growth Chart (16). Infants were excluded if their medical history included early-onset sepsis, major congenital or chromosomal abnormalities known to affect growth, liver failure, peri/intraventricular hemorrhage, if they had a sibling with diagnosed allergies or intolerances to lactose or cow's milk, or if they had severe respiratory distress after birth. The infants who were enrolled into the follow-up study had two or three visits depending on age at enrollment: at enrollment, 18 months CA, and 24 months CA. The timing of the cognitive assessments at 18 and 24 months aligned with routine check-up visits and reflected the period at which developmental delays may first become evident. Statistical analyses for the follow-up study are presented at 24 months CA since only two infants enrolled at 18 months CA.

### Study formulas and schedule of administration

Enrolled infants received a sequentially administered staged feeding system consisting of Stage 1 (LPF1, Nestlé, Switzerland) followed by Stage 2 (LPF2, Nestlé, Switzerland) liquid preterm formulas (Supplementary Figure 1 and Table 1). LPF1 provided 80 kcal/100 ml and 3.6 g intact protein/100 kcal, meeting the latest European guidelines for feeding preterm infants up to 1,800 g (8, 17, 18). LPF2 provided 73 kcal/100 ml and 2.8 g intact protein/100 kcal and aligned with nutrient needs of larger preterm infants (9, 13). Both formulas were enriched with structured lipids (sn-2 palmitate) and medium chain triglycerides for improved fat absorption and as accessible sources of energy (19). After completing 24 h of successful trophic feeding, LPF1 was initiated on pre-full enteral feeding day 1 (Pre-FEF D1) and the feeding volume gradually increased, as per standard practice. The first day of enteral feeding (FEF D1) was defined as the cessation of parenteral nutrition and a minimum enteral intake of 150 ml/kg/day. Administration of LPF1 continued through FEF D1 until reaching a body weight of 1,800 g, followed by LPF2 administration until 30 days post-discharge (30-d PD).

### Interventional study outcome measurements

#### Weight gain velocity and anthropometry

The primary outcome was WGV in g/kg/day from FEF D1 to the day 1,800 g body weight was achieved (20). Gains in weight, length, and head circumference (HC) were reported from Pre-FEF D1, through FEF D1, to 1,800 g (LPF1 period), and from 1,800 g to 30-d PD (LPF2 period). The corresponding anthropometric *z*-scores for weight-for-age (WAZ), length-for-age (LAZ), and head circumference-for-age (HCAZ) were calculated using the Fenton growth standards for preterm infants (21). All of the above measurements were also attained at 24 months CA in the follow-up study and are presented together with measurements from the interventional trial. Anthropometric *z*-scores at 24 months CA were calculated using the World Health Organization (WHO) Child Growth Standards as the reference population (22) and adjusted for CA (23). Details about the obtainment of each measurement are provided in Supplementary Methods.

#### Biomarkers of protein and bone health status

Serum markers of protein status [albumin and blood urea nitrogen (BUN)] as well as serum markers (alkaline phosphatase, phosphorus, calcium, sodium, and vitamin D) and urinary markers (calcium-to-creatinine ratio, phosphorus-to-creatinine ratio, and calcium-to-phosphorus ratio) of bone health were obtained on a weekly basis until the day the infant reached 1,800 g, in line with the NICU practice, and at 30-d PD. Tubular reabsorption of phosphate (TRP) was calculated as [1−(urine phosphate/plasma phosphate) × (plasma creatinine/(urinary creatinine × 1000))].

#### Gastrointestinal tolerance and adverse events

Gastrointestinal (GI) tolerance assessments, including gastric residual volumes (GRV), stool consistency (1 = watery, 2 = runny, 3 = mushy soft, 4 = formed, 5 = hard), stool frequency, abdominal distention, bloody stools, ballooning, regurgitation, crying, and sleep quality were logged in a daily diary for three consecutive days on a weekly basis during the NICU stay. After discharge, parents were provided a daily diary to record feeding intake, GI symptoms, and related behaviors for three consecutive days before the 30-d PD visit. AEs were recorded only for the interventional study and assessed for seriousness, duration, frequency, intensity, and relationship to study formulas from enrollment until 30 days after last formula intake.

### Follow-up study outcome measurements

#### Cognitive development

The main outcome of interest was the proportion of subjects with a BSID-III (15) cognitive score >70 at 24 months CA, a commonly used cut-off score for detecting developmental delays (24). The BSID-III is a widely used, standardized cognitive assessment of five neurodevelopmental domains: cognition, language, motor development, social–emotional, and adaptive behavior. Secondary endpoints included: (1) composite scores on the BSID scales; (2) parent-reported achievements of specific age-appropriate milestones using the Centers for Disease Control and Prevention (CDC) Milestone checklist (25); and (3) three sub-scores of infants’ temperament using the short version of the Early Child Behaviour Questionnaire (ECBQ-SF) (26), reflecting *surgency*, *negative affect*, and *effortful control*. The CDC checklist is a brief assessment tool that asks about how the child plays, learns, speaks, acts, and moves to provide indications on whether the child is developing normally in areas of social/emotional, language/communication, learning, and movement/physical development. The ECBQ-SF is validated among 1–3 years of age children and designed to measure temperament, including individual differences in emotional, motoric, and attentional reactivity and self-regulation. For details on the scoring systems of the tools, please see Supplementary Methods.

#### Hospitalization, healthcare usage, and feeding practice

Infants’ parents completed the Healthcare usage/Hospitalization and Feeding Practice Questionnaire, which was developed internally and collects data on feeding practices, healthcare usage, and hospitalizations between 30 days post-discharge and 24 months CA. Parents reported the number of healthcare usages and hospitalizations, as well as the reason for each hospitalization. Feeding information collected comprised types of nutrition received since hospital discharge, including type of formula, age at introduction of complementary foods, and consumption of other beverages in toddlerhood (i.e., cow's milk, growing up milk, and fortified milks).

### Statistical analysis

The sample size calculation for the interventional study was based on an expected WGV of 17.3 g/kg/day and greater than the minimally required intrauterine growth rate of 15 g/kg/day (20), or equivalently, a non-inferiority (NI) margin of −2.3 g/kg/day compared to WGV of the Fenton median infant of 17.3 g/kg/day at weeks 31–35. In total, 18 infants were required to demonstrate a WGV significantly greater than 15 g/kg/day, with standard deviation (SD) of 3.92 g/kg/day (27), alpha level of 0.05, and a design effect of 0.71. Enrollment of 30 infants was planned to account for up to 60% of subjects not meeting per protocol requirements. For the follow-up study, the maximum number of infants considered was that of infants enrolled in the interventional study.

**Figure 1:**
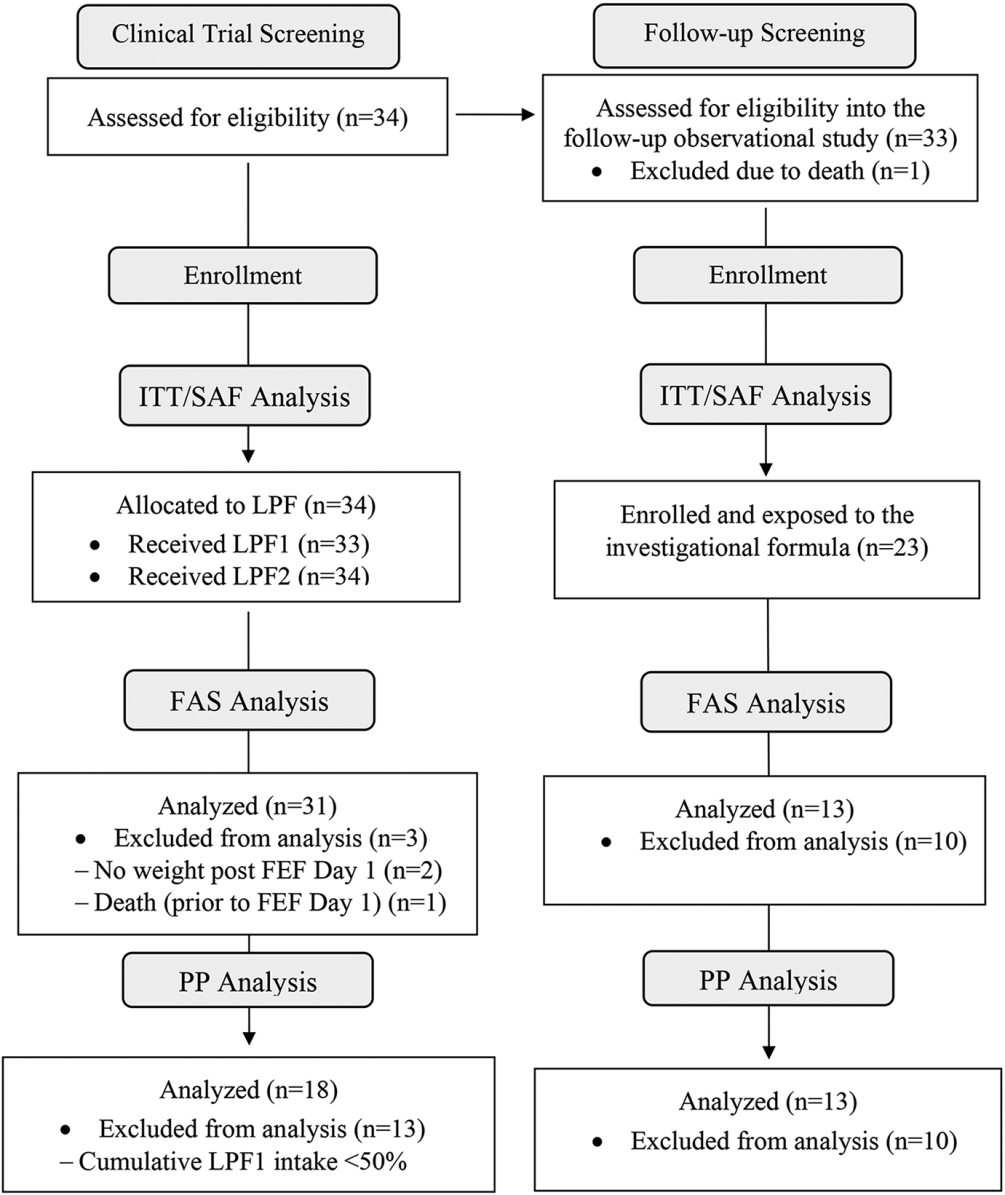
Subject disposition. The ITT analysis included all subjects enrolled into the study. The SAF included all infants exposed to at least one feeding of the investigational formula. The PP comprised all infants who received LPF1 at greater than 50% total enteral intake and who had no major protocol deviations. The FAS included all infants who received LPF1. FAS, full analysis set; FEF D1, day 1 of full enteral feeding; ITT, intention-to-treat set; LPF, liquid preterm formula; PP, per protocol set; SAF, safety analysis set.

In the interventional study, the intention-to-treat (ITT = 34) analysis included all subjects enrolled, while the safety analysis set (SAF = 34) included all infants exposed to at least one feeding of the investigational formula (Figure 1). The full analysis set (FAS = 31) included all infants who received LPF1 and had a primary endpoint measured. Infants in FAS who received LPF1 at greater than 50% of total enteral intake and who had no major protocol deviations comprised the per protocol set (PP = 18). The same definitions were used for ITT (*n* = 23), SAF (*n* = 23), PP (*n* = 13), and FAS (*n* = 13) populations in the follow-up study. Statistical analyses and results are presented for ITT and PP populations throughout, with the exception of main outcomes, for which PP and FAS were presented.

Weight gain velocity was computed using the exponential model as described by Patel et al., which is a validated and robust method for measuring growth velocities for LBW infants (28). Non-inferiority would be demonstrated if the lower bound of the 95% confidence interval (CI) for WGV was above 15 g/kg/day. To control for type I error, an ordered comparison was used to analyze the primary endpoint against 15 g/kg/day first for NI and then to 17.3 g/kg/day for superiority only if NI was demonstrated. Gains in weight, length, and HC, as well as changes in anthropometric *z*-scores, were computed and summarized using descriptive statistics. Statistical comparisons in growth were made between FEF D1 and 30-d PD using a linear mixed model adjusting for birth *z*-score, sex, and wGA. Mean ± SD values over three consecutive days were calculated for GI tolerance and GI-related behaviors. Time to event analysis using the Kaplan–Meier estimate (29) was done for the following events from birth to: FEF D1, day reaching 1,800 g, day to initiate minimal oral feeding, and day reaching full oral feeding.

As for cognitive development, percentage of infants with a BSID-III cognitive composite score >70 was calculated. The BSID-III composite score and subscales, CDC checklist scores, and ECBQ sub-scores were examined descriptively. Associations of growth measures, such as gains in weight, height, and HC, with composite scores on the BSID-III scales were examined using Spearman correlations, partial Spearman correlations, and linear regression. Partial Spearman correlations and linear regression models were adjusted for the following covariates: weight at birth, sex, and breastfeeding duration. Healthcare usage, number of hospitalizations, and feeding practice information, such as the type of nutrition received, were summarized descriptively. All AEs were coded using MedDRA version 21.0. *p*-values <0.05 were considered statistically significant. Analyses were conducted using SAS® Life Science Analytics Framework v5.4.1 (SAS Institute Inc., Cary, NC, USA).

## Results

### Subject characteristics and study formula intake

Thirty-four infants, with a mean ± SD postnatal age of 4.4 ± 1.9 days, were enrolled in the interventional study (Table 1). Infants ranged in wGA from 27 weeks and 2 days to 31 weeks and 6 days. Mean ± SD birth weight was 1,295.9 ± 152.8 g, and 85.3% were born by cesarean delivery. Twenty-three of those infants (10 female), with a mean CA at enrollment of 23.7 ± 1.9 months (range 18, 26), were enrolled into the follow-up study.

**Table 1:** Infant and maternal demographics of enrolled population for the interventional study and the follow-up study.

Interventional study	
Infant characteristics	*N* = 34
Gestational age (weeks), mean ± SD	30.0 ± 1.3
Gestational age classification, *n* (%) <28 weeks (extremely preterm) ≥28 and ≤32 weeks (very preterm)	1 (2.9) 33 (97.1)
Birth weight (g), mean ± SD	1,295.9 ± 152.8
Birth length (cm), mean ± SD	38.4 ± 2.6
Birth head circumference (cm), mean ± SD	27.6 ± 1.5
Cesarean delivery, *n* (%)	29 (85.3)
APGAR score (5 min), median (Q1, Q3)	7 (7, 8)
Male sex, *n* (%)	16 (47.1)
Caucasian ethnicity, *n* (%)	32 (94.1)
Postnatal age at enrollment (days), mean ± SD	4.4 ± 1.9
Maternal characteristics	*N* = 34
Maternal age at infant birth (years), mean ± SD	28.3 ± 5.0
Pre-pregnancy weight (kg), mean ± SD	62.5 ± 16.8
Pre-pregnancy BMI, mean ± SD	23.2 ± 5.7
Smoked during pregnancy, *n* (%)	2 (5.9)
Former smoker, *n* (%)	5 (14.7)
Follow-up study	
CA at enrollment, months	
Mean ± SD	23.7 ± 1.9
Median (min, max)	24 (18, 26)
Age category at enrollment	
18 months CA, *n* (%)	2 (8.7)
24 months CA, *n* (%)	21 (91.3)
Male sex, *n* (%)	13 (56.5)

APGAR, appearance, pulse, grimace, activity, and respiration; BMI, body mass index; CA, corrected age.

From FEF D1 to the day reaching 1,800 g, subjects in PP received an average of 146 ± 42.9 ml/kg/day of LPF1, whereas the mean intake in ITT was 89 ± 75.7 ml/kg/day. The intake of human milk was much lower, averaging 22.1 ± 28.8 ml/kg/day in PP and 79.1 ± 78.9 ml/kg/day in ITT. Intake of LPF2 from 1,800 g until 30-d PD was generally less, averaging 54.9 ± 53.1 ml/kg/day in PP and 45.2 ± 49.1 ml/kg/day in ITT. On average, subjects received at least one daily vitamin D supplementation for 9.7 days in PP and 13 days in ITT in the LPF1 period, and 3.3 days in PP and 4.4 days in ITT in the LPF2 period.

### Interventional study

#### Growth

**Figure 2:**
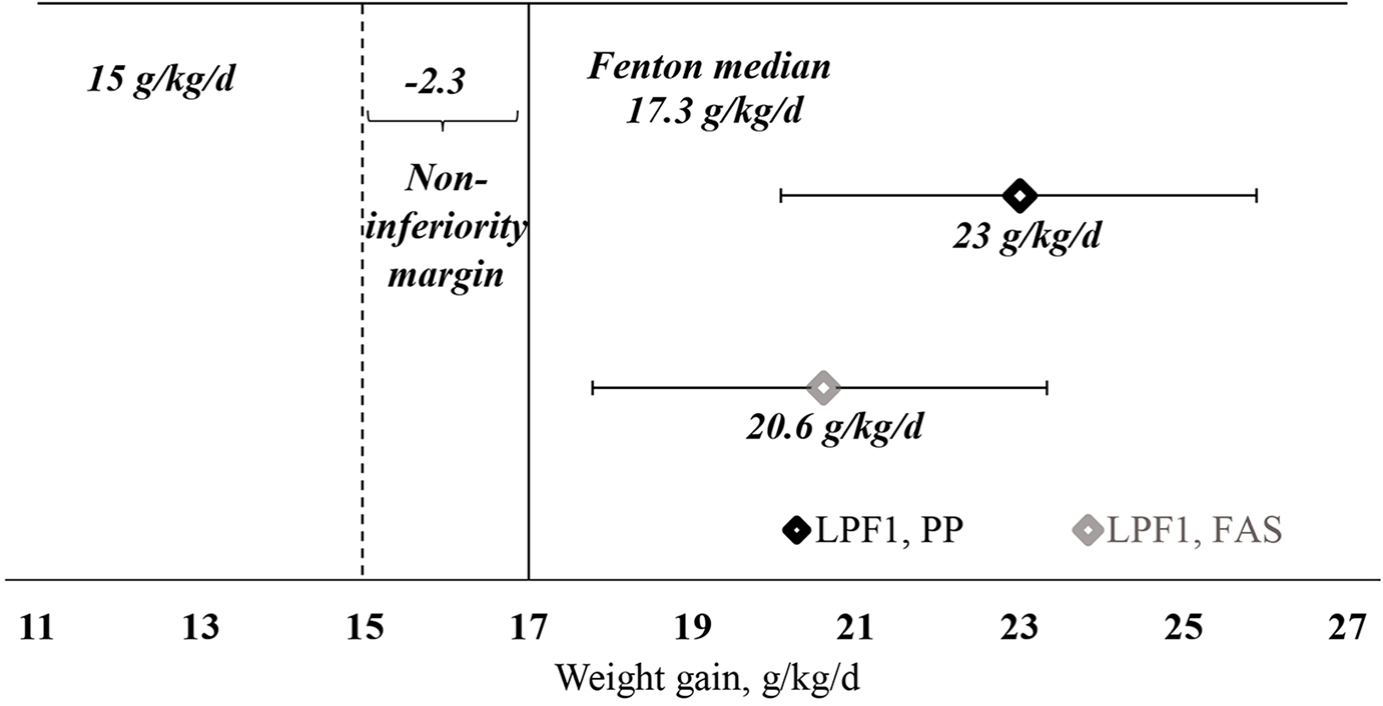
Weight gain velocity (g/kg/day) between FEF D1 and day reaching 1,800 g (LPF1 period), PP and FAS. The results are LS mean (95% CI) estimates from mixed model repeated measures adjusted for sex, gestational age, visit, site, and weight-adjusted weight gain between pre-FEF D1 and FEF D1. *p*-values: *p* < 0.001 (for PP and FAS) vs. non-inferiority margin. FAS, full analysis set; FEF D1, day 1 of full enteral feeding; LPF, liquid preterm formula; PP, per protocol set.

The mean adjusted WGV (95% CI) between FEF D1 and the day reaching 1,800 g (the period during which infants were fully enterally fed LPF1 at 150 ml/kg/day) was 23.0 (20.1–25.9) g/kg/day in PP and 20.6 (17.8–23.3) g/kg/day in FAS (Figure 2). In both analysis sets, the lower bound of the 95% CI (20.1 and 17.8 g/kg/day in PP and FAS, respectively) was greater than the prespecified minimal WGV of 15 g/kg/day (*p* < 0.001 for PP and FAS) and greater than the prespecified expected WGV of 17.3 g/kg/day (PP: *p* < 0.001; FAS: *p* = 0.012).

From FEF D1 to the day reaching 1,800 g with LPF1, gains in weight and length met or exceeded the recommended 10–30 g/day and 1 cm/week respective goals (16), and were sustained with LPF2 and length until 30-d PD (Table 2). Gains in HC also met recommended goals (i.e., 0.9–1 cm/week) (16) from FEF D1 to the day reaching 1,800 g with LPF1, after which they stabilized around 0.8 cm/week with LPF2. Median time (95% CI) in days from birth to: (i) FEF D1 was 9 (8–10) (PP and ITT); (ii) regain birth weight was 13 (8–15) (PP) and 14 (9–15) (ITT); (iii) day reaching 1,800 g was 23 (19–29) (PP) and 27.5 (23–31) (ITT); (iv) day in which minimal oral feeding was initiated was 19.5 (14–23) (PP) and 22 (19–23) (ITT); (v) the day in which full oral feeding was reached was 49.5 (38–55) (PP) and 51.5 (50–59) (ITT); and (vi) hospital discharge was 48.5 (38–55) (PP) and 51.5 (49–59) (ITT).

**Table 2:** Anthropometric gains from FEF D1 to the day reaching 1,800 g and to 30-d PD for ITT population (*N* = 34) and PP population (*N* = 18).

	FEF D1 to 1,800 g Mean ± SD		FEF D1 to 30-d PD Mean ± SD	
	ITT (*N* = 31)	PP (*N* = 18)	ITT (*N* = 32)	PP (*N* = 18)
Weight gain (g/day)	31.6 ± 9.4	37.2 ± 8.3	34.7 ± 5.9	36.2 ± 6.6
Length gain (cm/week)	1.2 ± 1.0	1.5 ± 1.2	1.1 ± 0.2	1.2 ± 0.2
Head circumference gain (cm/week)	0.9 ± 0.4	0.9 ± 0.4	0.8 ± 0.1	0.8 ± 0.1

FEF D1, day 1 of full enteral feeding; ITT, intention-to-treat set; PD, post-discharge; PP, per protocol set.

**Figure 3:**
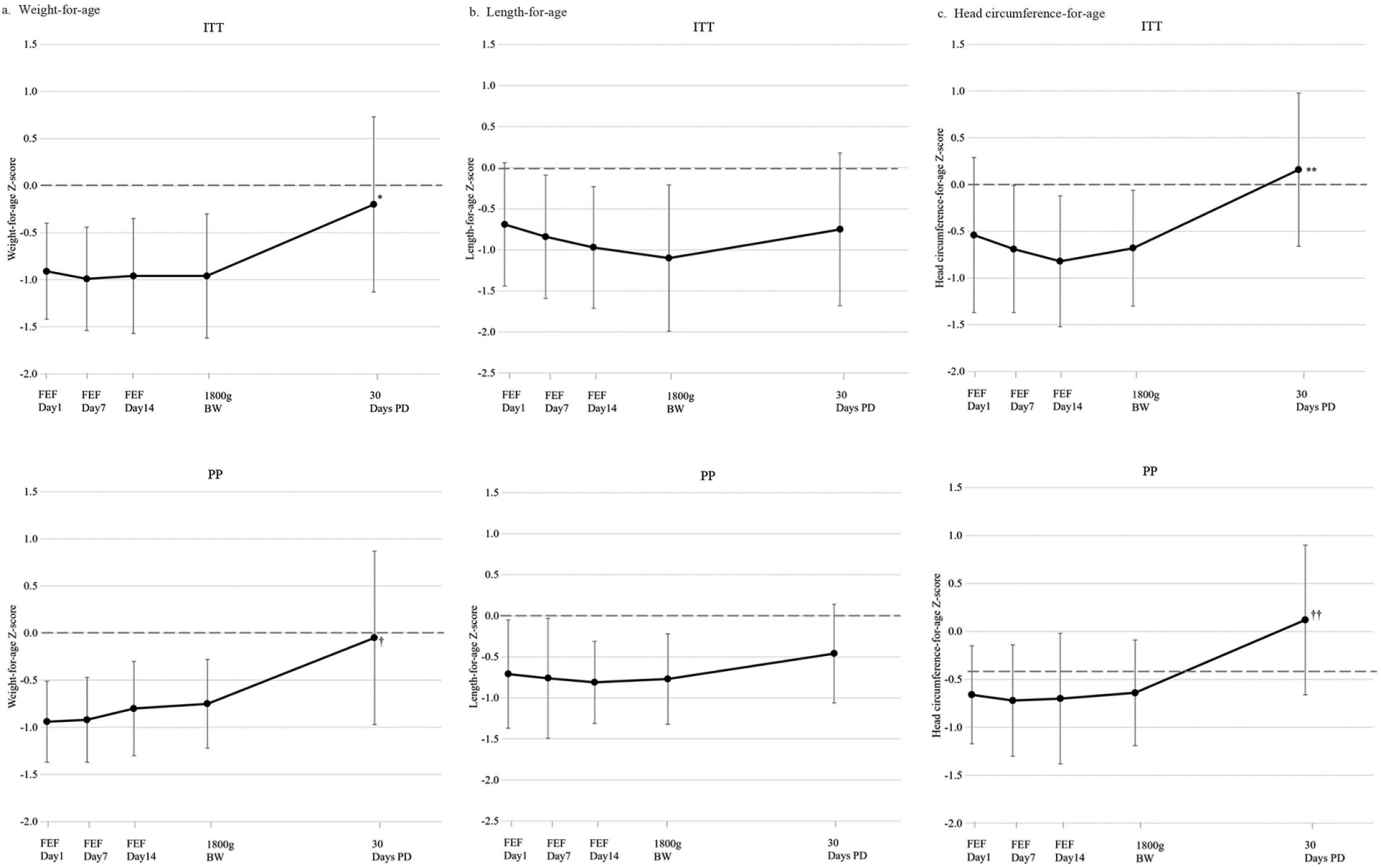
Anthropometric Fenton *z*-scores for weight-for-age **(A)**, length-for-age **(B)**, and head circumference-for-age **(C)** in ITT (top) and PP (bottom) populations. Mean ± SD *z*-scores calculated using Fenton preterm growth charts (16). Comparisons in growth between FEF D1 and 30-d PD were made using a linear mixed model, adjusting for birth *z*-score, sex, and wGA. Comparisons with 24-month CA *z*-scores are not presented as they were calculated using the WHO Child Growth Standards as the reference population (22). *p < 0.001 vs. FEF D1; ^†^*p* = 0.02 vs. FEF D1; **p = 0.001 vs. FEF D1; ^††^*p* = 0.02 vs. FEF D1. BW, body weight; FEF, full enteral feeding period; ITT, intention-to-treat set; PD, post-discharge; PP, per protocol set.

In general, trends in WAZ, LAZ, and HCAZ in the PP and the ITT sets were very similar (Figure 3). From the day of reaching 1,800 g to 30-d PD, WAZ in both PP and ITT progressed toward the Fenton median (Figure 3A). Similar trends were seen for LAZ and HCAZ, with LAZ progressing toward the Fenton median by 30-day PD and HCAZ exceeding the Fenton median by 30-d PD in both PP and ITT (Figures 3B,C**)**. At 24 months, WAZ were slightly below the WHO growth standard median, whereas LAZ and HCAZ exceeded it. Between FEF D1 and 30-d PD, there was a significant increase in WAZ (*p* = 0.02 in PP; *p* < 0.001 in ITT) and HCAZ (*p* = 0.02 in PP; *p* = 0.001 in ITT), but not in LAZ (*p* = 0.617 in PP; *p* = 0.651 in ITT).

#### Gastrointestinal tolerance and adverse events

Mean stool frequency ranged from 2.5 to 3.3 stools per day. Stool consistency was very close to 3 (mushy soft) at all time points (range 2.8–3.1), with a mean ± SD score of 2.9 ± 0.4 on the day reaching 1,800 g and 2.9 ± 1.1 at the 30-d PD visit. The mean GRV was 0.8 ml/kg during the pre-FEF period and further dropped to 0 by FEF D21, which was sustained until discharge. Bloody stools were experienced by one infant (2.9%) in the pre-FEF period and two infants at discharge (5.9%). During the 30-d PD period, 9 (26.5%) infants had stomach ballooning and 10 (29.4%) infants experienced more than two regurgitations per day (range 0–8 times per day). In addition, crying more than 1 h per day was reported by 9 infants (26.5%) and 11 (32.6%) infants woke up more than three times at night. All subjects experienced at least one AE during the study period (from enrollment until 30 days after the last day of product intake), although the majority were mild (*N* = 14; 41.2%) and moderate (*N* = 15; 44.1%). Four subjects (11.8%) experienced a serious adverse event (SAE) during the LPF1 period (two experienced necrotizing colitis and two experienced sepsis) and two subjects (5.9%) experienced SAEs during the LPF2 period (one experienced ileus paralytic and another experienced meningitis). None of the AEs that occurred was classified as related to the study formulas LPF1 and LPF2.

#### Biochemical assessment of protein status and bone health

In ITT, the protein markers BUN and albumin were within the normal clinical ranges (30, 31) from FEF D1 to 1,800 g and through the 30-d PD period (Table 3). From the markers of bone health, calcium and TRP values were stable and within normal clinical ranges throughout the study duration. Median serum alkaline phosphatase concentration was within the normal range: it was slightly elevated at FEF D1 [median (range): 369.6 (288.0–494.4) U/L], after which levels declined and remained lower from FEF D7 onwards. Median serum phosphorus levels were above the lower reference threshold of 1.5 mmol/L for hypophosphatemia and remained stable at 2.1–2.2 mmol/L throughout the study. Median (range) serum vitamin D levels were low at FEF D1 and day reaching 1,800 g [21.3 (12.0–37.6) and 20.8 (17.1–48.1) ng/ml, respectively], but doubled by 30-d PD [45.5 (34.5–68.9) ng/ml]. Trends of changes in markers of protein and bone health were very similar in PP (Supplementary Table 2) and within normal clinical ranges.

**Table 3:** Biomarkers of protein status and bone health between FEF D1 and 30-d PD for ITT population (*N* = 34).

	Study day				
	FEF day 1	FEF day 7	FEF day 14	1,800 g	30-d PD
	Median (Q1, Q3) *n*	Median (Q1, Q3) *n*	Median (Q1, Q3) *n*	Median (Q1, Q3) *n*	Median (Q1, Q3) *n*
Biomarkers of protein status					
Serum albumin (g/L)	36.1 (33.4–38.5) 28	33.5 (31.5–36.9) 31	32.4 (30.7–35.8) 30	31.6 (30.8–33.5) 30	35.4 (33.6–37.2) 31
Serum BUN (mmol/L)	6.2 (4.4–7.3) 29	4.1 (3.1–5.0) 31	3.4 (1.9–3.7) 31	2.0 (1.6–4.0) 30	4.2 (2.6–4.7) 32
Biomarkers of bone health					
Serum alkaline phosphatase (U/L)	369.6 (288.0–494.4) 27	312.0 (245.4–406.8) 31	276.0 (236.9–461.4) 31	296.7 (245.4–448.2) 30	331.8 (258.6–380.7) 32
Serum creatinine (mmol/L)	57.2 (47.0–65) 29	46.1 (37.0–53) 31	37.0 (30.7–43.0) 31	31.5 (29.7–37.0) 30	17.0 (16.0–20.3) 32
Serum phosphorus (mmol/L)	2.1 (1.9–2.4) 29	2.1 (2.0–2.3) 31	2.1 (1.9–2.3) 31	2.1 (2.0–2.2) 30	2.2 (2.1–2.4) 32
Serum calcium (mmol/L)	2.6 (2.4–2.6) 29	2.6 (2.5–2.6) 31	2.5 (2.4–2.5) 31	2.5 (2.4–2.6) 30	2.6 (2.5–2.6) 32
Serum sodium (mmol/L)	139 (137–141) 29	138 (136–139) 31	138 (136–139) 31	138.1 (136–140) 30	138 (137–139) 32
Serum 25(OH)D (vitamin D) (ng/ml)	21.3 (12.0–37.6) 31	^a^	^a^	20.8 (17.1–48.1) 30	45.5 (34.5–68.9) 30
Urinary calcium/creatinine ratio (mmol/L)	0.4 (0.2–0.7) 29	0.9 (0.5–1.5) 30	0.8 (0.5–1.2) 30	0.7 (0.5–1.6) 30	1.4 (0.4–4.4) 27
Urinary phosphorus/creatinine ratio (mmol/L)	5.8 (3.0–11.5) 28	6.6 (2.4–8.9) 30	5.3 (2.6–7.4) 31	5.0 (3.0–8.4) 30	6.8 (4.3–10.0) 30
Urinary calcium/phosphorus ratio (mmol/L)	0.1 (0.0–0.3) 28	0.1 (0.1–0.6) 31	0.1 (0.1–0.4) 30	0.2 (0.1–0.5) 30	0.2 (0.1–0.4) 30
TRP (%)	90 (80–90) 28	90 (80–90) 30	90 (90–100) 31	90 (90–100) 30	90 (90–100) 30

BUN, blood urea nitrogen; FEF day 1, full enteral feeding day 1; ITT, intention-to-treat set; TRP, tubular resorption of phosphate.

^a^ Per study protocol, 25(OH)D vitamin D not drawn at FEF D7 or FEF D14 to spare excessive blood collections.

### Two-year follow-up

#### Cognitive development and temperament

The mean ± SD scores for the cognitive scale of the BSID were 97.3 ± 13.9 (PP) and 103.3 ± 13.0 (ITT), close to the standardized mean ± SD of 100 ± 15 (Table 4). All subjects had cognitive scores >70. Similar scores were observed for the rest of the BSID scales. Results from the CDC Milestone checklist revealed the highest scores for movement/physical development (82.0 ± 15.6 in PP; 84.2 ± 13.6 in ITT) and language/communication (82.1 ± 17.3 in PP; 84.1 ± 17.8 in ITT), followed by scores for social/emotional (75.4 ± 24.7 in PP; 80.0 ± 22.6 in ITT) and cognitive (67.3 ± 21.4 in PP; 73.9 ± 19.6 in ITT) developmental areas (Supplementary Table 3). Assessment of temperament showed relatively high mean scores for surgency (4.8 ± 0.7 in PP; 4.8 ± 0.5 in ITT) and effortful control (5.1 ± 0.6 in PP; 5.2 ± 0.6 in ITT), and relatively low mean scores for negative affect (3 ± 0.6 in PP; 3.1 ± 0.5 in ITT) (Supplementary Table 4).

**Table 4:** Proportion of preterm infants above the 70-score cut-off for each composite score of the BSID-III at 24 months CA for ITT population (*N* = 23) and PP population (*N* = 13).

Composite score	ITT (*N* = 23)		PP (*N* = 13)	
	Score >70 *N* (%)	Mean ± SD Median (range)	Score >70 *N* (%)	Mean ± SD Median (range)
Cognitive^a^	23 (100%)	103.3 ± 13.0 105 (95–110)	13 (100%)	97.3 ± 13.9 95 (90–100)
Language^b^	23 (95.6%)	90.7 ± 14.7 91 (68–141)	12 (92.3%)	84.1 ± 10.1 83 (68–103)
Motor development^b^	23 (100%)	97.4 ± 12.9 94 (73–133)	13 (100%)	92.4 ± 8.5 94 (73–107)
Social-emotional^b^	23 (100%)	109 ± 20.6 110 (75–145)	13 (100%)	102.4 ± 21.8 105 (75–145)
Adaptive behavior^b^	23 (100%)	101.5 ± 15.5 99 (73–126)	13 (100%)	97.7 ± 14.4 95 (73–122)

CA, corrected age; ITT, intention-to-treat set; PP, per protocol set.

^a^ Main outcome of interest.

^b^ Secondary outcome.

Significant positive associations between gains in weight, length, and HC for the period FEF day 1 to 24 months CA and composite scores on BSID-III scales were revealed consistently in Spearman and partial Spearman correlational and linear regression models, respectively, in the ITT set, as follows: significant positive association of weight gain with adaptive behavior (*p*-values: <0.001, 0.006, and 0.006) and motor development (*p*-values: 0.005, 0.003, and 0.008); significant positive association of length gain with adaptive behavior (*p*-values: <0.001, 0.006, and 0.007); and significant positive association of HC gain with motor development (*p*-values: 0.016, 0.037, and 0.042). Similar associations were revealed in the PP, although they did not reach statistical significance.

#### Hospitalization, healthcare usage, and feeding practice

In the period between 30-d PD and 24-month CA visits, 90% of infants had at least one healthcare consultation, most frequently with a pediatrician/general practitioner (92.3% in PP; 87% in ITT) (Supplementary Table 5). Mean ± SD hospital stay was 5.0 ± 2.1 days in the PP and 4.2 ± 2.4 days in the ITT group. Only one visit was due to an infection in each analysis set. Seven (53.9%) and 13 (56.5%) infants received LPF2 formula in the PP and ITT sets, respectively (Supplementary Table 6). Infants were introduced to complementary foods around a median (range) age of 6 (4–12) months. Consumption of other beverages included fortified cow's milk, regular cow's milk, growing up milk, and other types of milk.

## Discussion

In a prospective, open-label, multicenter interventional trial coupled with a 24-month follow-up observational study, our findings demonstrated that a new two-stage feeding system for preterm infants, composed of Stage 1 and Stage 2 formulas designed to provide tailored nutrition during hospital stay and after discharge in line with recent expert recommendations, supported adequate growth patterns, good GI tolerance, a safe profile of nutritional biomarkers, and age-appropriate neurodevelopmental outcomes in VLBW preterm infants.

Our research demonstrated non-inferiority of mean adjusted weight gain velocity with LPF1 formula to the recommended intrauterine growth rates (16), with weight gain in both PP and FAS analysis sets (23.0 and 20.6 g/kg/day, respectively) well above 17.3 g/kg/day and accompanied by gains in length (1.2–1.5  cm/week) and HC (0.9–1.0 cm/week) at 1,800 g body weight that met or exceeded Fenton growth standards (16). Age-appropriate growth patterns were supported continuously with LPF2 after hospital discharge, with gains in weight (35–36 g/day) and length (1.1–1.2 cm/week) meeting or exceeding recommended goals, and HC gains stabilizing at around 0.8 cm/week. We observed comparable gains in length and HC and higher weight gain with our LPF1 formula in relation to premature infants of similar post-menstrual age fed high-protein content (3.4–3.6 g/100 kcal) in Vietnam (length gain 1.2 cm/week, HC gain 0.9 cm/week, weight gain 17 g/kg/day) (32) and in Western Europe (length gain 1.2 cm/week, HC gain 1.0 cm/week, weight gain 18.3 g/kg/day) (27). Importantly, our findings add to the body of evidence supporting the effect of higher protein levels on promoting adequate growth in preterm infants (10, 13, 33). Collectively, evidence on energy and protein fortification in both formula and human milk was also deemed “suggestive” for a significant impact on growth during hospitalization in LBW infants in a recent umbrella review of systematic reviews and meta-analyses (34). Complementing and building on these findings, in a Cochrane review, Fenton et al. demonstrated that higher (3.0–4–0 g/kg/day) compared to lower (<3.0 g/kg/day) protein intake during hospital stay supported greater weight gain (∼2 g/kg/day) (35). Studies on the effect of post-discharge protein-enriched formulas in preterm infants have been less conclusive, with some interventional trials showing significantly improved growth parameters (36, 37) and others showing mixed findings (38, 39) or a lack of a significant benefit (40, 41). Nonetheless, when these inconsistences were addressed in a systematic review with evidence mapping by Teller et al., they were shown to be attributed to the high heterogeneity in study design of previous interventions, and the results revealed increased lean mass accretion and HC growth with higher protein-to-energy ratios (≥2.5–3.0) (13).

In the present study, albumin and BUN levels remained elevated at the beginning of the protein supplementation, after which they progressively declined and stabilized within normal ranges by the time infants reached 1,800 g and throughout post-discharge. Although data from these two biomarkers alone do not present a complete assessment of protein status (42), they suggest an improved nitrogen balance at the early stage of formula administration (with LPF1), as well as sufficient energy availability for protein utilization with lower levels of protein post hospitalization (with LPF2) (BUN levels remained higher than the 1.6 mmol/L cut-off for insufficient protein intake) (43). Furthermore, the lack of signs of metabolic stress combined with better growth altogether indicate safe high-protein levels in our formulas in preterm infants. Evaluation of bone mineralization status was another crucial component of this research due to the high prevalence of bone metabolic disease (BMD) in premature infants (44). Our findings present evidence of a satisfactory bone mineral status, with mean serum levels of calcium and phosphorus within normal ranges and stable across both LPF1 and LPF2 periods, and serum alkaline phosphatase steadily decreasing over time. Together, serum alkaline phosphatase and phosphorus are known to be excellent biochemical markers of BMD, yielding a specificity of 70% and a sensitivity of 100% (45). Importantly, we do not report cases of hypophosphatemia. These results are in contrast with findings from previous studies whereby higher protein intakes in VLBW infants were consistently associated with hypophosphatemia (46–48). Indeed, there has been growing evidence to propose multiple physiological mechanisms believed to account for an additional phosphorus demand in formula-fed premature infants, such as an increased cellular uptake of phosphorus following high-protein intake and a rise in phosphorus levels through an increased calcium absorption in the presence of sn-2 palmitate (47–49). In the current study, serum levels of phosphorus and alkaline phosphatase provided evidence in favor of phosphorus adequacy. Taken together, these results and the levels of nutrients such as calcium, vitamin D, and phosphorus in our two-staged formulas, designed to meet the latest guidelines and nutritional recommendations for preterm infants (8, 18), suggest the provision of an adequate and safe nutritional status, which may support bone mineralization and the prevention of postnatal osteopenia (50).

Findings from the follow-up observational study indicated age-appropriate neurocognitive development at 24 months with more than 96% of infants achieving BSID-III composite scores within normal ranges (51). To our knowledge, this is the first study in VLBW infants fed protein- and nutrient-enriched formulas to have evaluated developmental milestones and infant temperament. Scores on all of the domains on the CDC checklist assessing developmental milestones were relatively high (>65 on cognitive domain and >80 on language, movement, and emotional domains). In addition, the ECBQ sub-scores assessing temperament were not only found to be similar to those previously reported in preterm populations (52), but the scores may also be indicative of high activity and positive affect, emotional stability, and self-awareness (53). Our results are consistent with previous studies of nutrient-enriched preterm formulas to have found an improved cognitive performance at 18 months CA (54, 55) and at 24 months CA (56) as measured by BSID-III, as well as at 8 and 16 years of age as measured by other cognitive ability scales (57, 58). Furthermore, the positive association of gains in weight, length, and HC we observed with several BSID-III domains is in line with previous findings of studies whereby infant growth was significantly associated with better neurodevelopmental outcomes, assessed by BSID scores, at several sensitive postnatal periods: before term (59), after term (60), and in later childhood (61). Interestingly, neither early high energy intake nor high amino acid supplementation, respectively, were shown to impact neurodevelopment in preterm infants in previous systematic review (62) and meta-analysis (14), possibly due to the low number of studies and inconsistent methodological approaches used. In the last decade, there has been accumulating evidence on the role of *holistic* early nutrition in the process of myelination, the main mechanism through which brain plasticity and neural connectivity are believed to influence cognitive development (63, 64). Preterm birth is associated with delayed myelination, decreased interhemispheric functional connectivity at term equivalent age, and cerebral white matter injury (65–67). A recent clinical trial in neurotypical term infants demonstrated a significant effect of a nutrient blend containing sphingomyelin, docosahexaenoic acid (DHA), arachidonic acid (ARA), iron, vitamin B12, and folic acid on myelin structure, myelin volume, and rate of myelination at 3 and 6 months of life (68). Adding to this body of evidence, significant positive associations were also reported between myelination and dietary intakes of protein, fatty acids, iron, magnesium, copper, folic acid, selenium, and vitamins A, B1, B2, and B6 at 6–20 months (69). While more interventional studies specific to the preterm population are needed to draw conclusions, myelination, indeed, may have been the key mechanism through which some nutrients (sphingomyelin, DHA/ARA, and iron) with known individual impact on cognitive and behavioral outcomes of preterm infants in previous clinical trials (70, 71) might as well have supported the neurodevelopment of preterm infants fed a nutrient-enriched two-stage feeding system in our study.

Main strengths of this research are: documenting the effect of an early nutritional intervention in preterm infants using multiple growth parameters, biomarkers, GI tolerance, and several well-established cognitive scales; the use of multiple clinical sites in two European countries for improved external validity; and the long-term follow-up on growth and neurodevelopmental outcomes. Key limitations include a small sample size, a single-arm design without a comparator, and the availability of cognitive data only at 24 months. Given the low statistical power and the inability to adjust for a more comprehensive list of covariates in our statistical analyses, we also acknowledge the risk of residual confounding. Predetermined strategies to manage these limitations involved regular safety data monitoring by internal and external medical experts and clinicians, evaluation of growth rates against validated intrauterine growth standards, examination of nutritional biomarkers against established reference values, and assessment of neurodevelopmental outcomes using standardized tests. Importantly, our findings pertain to VLBW preterm infants (27–32 wGA; birth weight ≤1,500 g) and conclusions should, therefore, be inferred with caution for extremely preterm (<28 weeks gestation) and moderate-to-late preterm infants (32–37 weeks gestation).

## Conclusion

Our two-stage preterm formulas were shown to support postnatal gains of weight, length, and HC among VLBW infants, consistently meeting desired intrauterine growth goals, while maintaining adequate growth during the post-discharge follow-up period. Results also indicated that both formulations are safe, well-tolerated, and support cognitive development within normal ranges.

## Data Availability

The original contributions presented in the study are included in the article/Supplementary Material, further inquiries can be directed to the corresponding author.
