# Supplementary material for: Effects of two-stage preterm formulas on growth, nutritional biomarkers, and neurodevelopment in preterm infants
Source: Front Pediatr. 2024 Nov 22;12:1427050. doi: 10.3389/fped.2024.1427050 (PMC11620880; doi:10.3389/fped.2024.1427050)
Supplement: Supplementary file 1 [file Datasheet1.pdf]

Supplementary Material

Methods

Study procedures

Anthropometry

Weight was measured daily from enrollment until hospital discharge inside the incubator. At full enteral feeding (FEF) day 1, 1800g body weight, 30-day post discharge (PD), and 24 months corrected age (CA), weight was measured using a calibrated electronic scale (Baby Scale 727, Seca, Semur-en-Auxois, France) to the nearest 1g outside the incubator. Measurements were taken until two measures agreed within 10g and then the average of those two measures was considered for data analysis. Length and head circumference (HC) measurements were obtained using a specialized length board and a standard nonelastic plastic-coated measuring tape placed over the occiput and supraorbital ridges, respectively. Both were obtained to the nearest 0.1cm weekly until FEF day 21, and at PD, 30-day PD, and 24 months CA. Length measurements were taken until two measures agreed within 0.2cm in the interventional study and within 0.5cm in the follow-up study, respectively. HC measurements were obtained until two measurements were within 0.2cm in both studies. As with weight measurements, the average of the two measures recorded for length and HC at each timepoint was considered for data analysis.

Cognitive development

At 24 months CA, a trained study member administered The Bayley Scales of Infant and Toddler Development — Third Edition (BSID-III)[1], which takes approximately 30-60 minutes for completion. In BSID-III, a composite score is calculated for each of the five neurodevelopmental scales. Composite scores are scaled to a metric, with a mean of 100, standard deviation (SD) of

15, and a range of 40 to 160. The Centers for Disease Control (CDC) Developmental Milestone Checklist assesses four developmental areas, for each of which a sub-score was calculated: social/emotional, language/communication, learning, and movement/physical development[2]. For each milestone, the proportion of ticked answers was summarized as a proportion varying from 0 (none of the responses for the milestone were chosen) to 100 (all of the responses for the milestone were chosen). Finally, child temperament was evaluated using the short version of the Early Child Behaviour Questionnaire (ECBQ-SF) which takes approximately 10-15 minutes to complete with each question rated on a 7-point Likert scale ranging from 1=extremely untrue to 7=extremely true[3]. The three sub-scores examined consisted of the following components: *surgency* - impulsivity, activity level, high-intensity pleasure, sociability, and positive anticipation; *negative affect* - discomfort, fear, motor activation, sadness, perceptual sensitivity, shyness, soothability, and frustration; and *effortful control* - inhibitory control, attention shifting, low-intensity pleasure, cuddliness and attention focusing. All cognitive tests were translated in parents' native languages as follows: BSID-III was translated by NCS Pearson, Inc., a Minnesota corporation, with a Master License Agreement; CDC checklist and ECBQ-SF were translated by a private agency.

Supplementary Tables and Figures

Supplementary Figure 1. Trial design

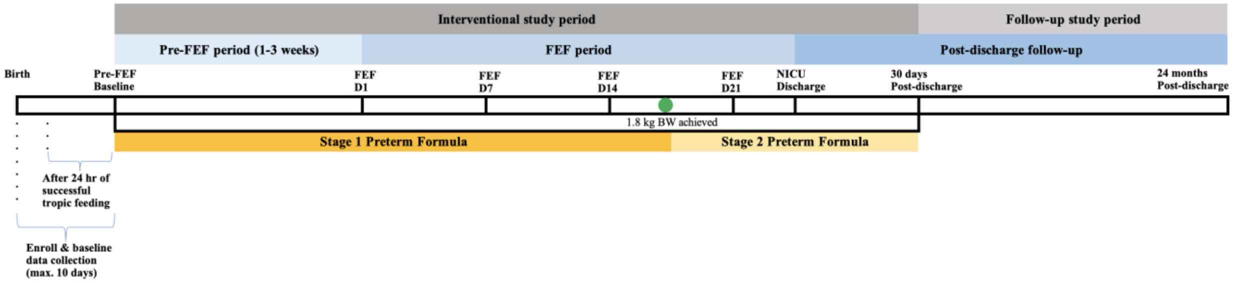

Abbreviations: FEF=Full enteral feeding; NICU=Neonatal intensive care unit

Definition of FEF is 150ml/kg/day of enteral feeding and discontinuation of parenteral feeding; Pre-FEF period is the duration between Pre-FEF Day 1; FEF day 1 denotes end of parenteral nutrition and minimum enteral intake of 150ml/kg/day.

Supplementary Table 1. Selected macro- and micronutrient compositions of the study formulas

**Nutrient**	**Stage 1 LPF1** (per 100 kcal unless otherwise noted)	**Stage 2 LPF2** (per 100 kcal unless otherwise noted)
Energy (kcal/100 ml)	80	73
**Macronutrients**		
Protein (g)	3.6	2.8
Fat (g)	4.7	4.8
ARA (mg)	26.0	26.4
DHA (mg)	24.7	24.9
Carbohydrate (g)	10.8	11.4
**Micronutrients**		
Sodium (mg)	70.3	48.5
Potassium (mg)	140.6	129.7
Calcium (mg)	149.7	115.4
Phosphorus (mg)	95.0	74.1
Vitamin D (µg)	212	100

Abbreviations: ARA=Arachidonic acid; DHA=Docosahexaenoic acid; LPF=Liquid preterm formula

Supplementary Table 2. Biomarkers of protein status and bone health between FEF day 1 and 30-d PD, PP population (N=18)

	Study Day				
	FEF Day 1<br>Median (Q1, Q3)<br>*n*	FEF Day 7<br>Median (Q1, Q3)<br>*n*	FEF Day 14<br>Median (Q1, Q3)<br>*n*	1800 g<br>Median (Q1, Q3)<br>*n*	30-d PD<br>Median (Q1, Q3)<br>*n*
Biomarkers of Protein Status					
Serum albumin, g/L	36.1 (32.8, 38.5)<br>*15*	32.9 (30.9, 35.3)<br>*17*	32.1 (31.0, 33.6)<br>*16*	31.6 (30.9, 33.0)<br>*17*	35.4 (33.8, 37.2)<br>*17*
Serum BUN, mmol/L	5.4 (4.4, 7.2)<br>*16*	4.5 (3.7, 6.0)<br>*17*	3.6 (2.1, 3.7)<br>*17*	3.7 (2.1, 4.6)<br>*17*	4.5 (3.6, 4.8)<br>*18*
Biomarkers of Bone Health					
Serum alkaline phosphatase, U/L	329.1 (263.9, 369.6)<br>*14*	268.8 (241.2, 329.4)<br>*17*	261.0 (234.0, 345.6)<br>*17*	276.0 (245.4, 305.3)<br>*17*	325.6 (283.2, 359.4)<br>*18*
Serum creatinine, mmol/L	53.0 (44.5, 65)<br>*16*	46.1 (37.0, 52.8)<br>*17*	37.0 (29.9, 42.0)<br>*17*	31.7 (30.7, 37.4)<br>*17*	19.0 (18.0, 20.8)<br>*18*
Serum phosphorus, mmol/L	2.2 (1.9, 2.5)<br>*16*	2.2 (2.1, 2.4)<br>*17*	2.2 (2.0, 2.2)<br>*17*	2.2 (2.1, 2.3)<br>*17*	2.2 (2.2, 2.3)<br>*18*
Serum calcium, mmol/L	2.5 (2.4, 2.6)<br>*16*	2.5 (2.5, 2.6)<br>*17*	2.5 (2.4, 2.5)<br>*17*	2.5 (2.4, 2.6)<br>*17*	2.6 (2.5, 2.6)<br>*18*
Serum sodium, mmol/L	139.5 (137, 141.4)<br>*16*	138.0 (136, 140)<br>*17*	139.0 (136, 140)<br>*17*	138.1 (136, 139)<br>*17*	138.0 (136, 139)<br>*18*
Serum 25(OH)D (Vitamin D), ng/mL	25.2 (15.3, 58.6)<br>*17*	*	*	24.0 (18.3, 67.3)<br>*16*	45.5 (35.5, 69.2)<br>*18*
Urinary calcium/creatinine ratio, mmol/L / mmol/L	0.4 (0.2, 0.7)<br>*16*	0.3 (0.4, 1.7)<br>*17*	0.7 (0.4, 1.1)<br>*16*	0.7 (0.5, 1.1)<br>*17*	0.8 (0.5, 1.7)<br>*18*
Urinary phosphorus/creatinine ratio, mmol/L / mmol/L	9.6 (6.2, 12.7)<br>*15*	8.3 (2.4, 8.9)<br>*17*	6.1 (5.0, 8.9)<br>*17*	6.7 (4.7, 8.9)<br>*17*	7.2 (4.3, 10.0)<br>*18*
Urinary calcium /phosphorus ratio, mmol/L / mmol/L	0.0 (0.0, 0.1)<br>*15*	0.1 (0.1, 0.1)<br>*17*	0.1 (0.1, 0.2)<br>16	0.1 (0.1, 0.2)<br>*17*	0.1 (0.1, 0.4)<br>*18*
Tubular resorption of phosphate (TRP), %	80 (70, 90)<br>*15*	90 (80, 90)<br>*17*	90 (80, 90)<br>*17*	90 (90, 90)<br>*17*	90 (90, 100)<br>*18*

Abbreviations: BUN=Blood urea nitrogen; FEF day 1=Full enteral feeding day 1; PD=post discharge; PP=Per protocol set

* Per study protocol, 25(OH)D vitamin D not drawn at FEF Day 7 or FEF Day 14 to spare excessive blood collections.

Supplementary Table 3. Centrs for Disease Control and Prevention (CDC) Developmental Milestone Checklist at 24 months CA, ITT population (N=23) and PP population (N=13)

**CDC milestone sub-score**	**ITT (N=23) Mean±SD) Median [Range]**	**PP (N=13) Mean±SD Median [Range]**
Cognitive	73.9±19.6<br>75 [25, 100]	67.3±21.4<br>62.5 [25, 100]
Language / Communication	84.1±17.8<br>83.3 [50, 100]	82.1±17.3<br>83.3 [50, 100]
Movement / Physical development	84.2±13.6<br>85.7 [57.1, 100]	82±15.6<br>85.7 [57.1, 100]
Social / Emotional	80±22.6<br>80 [40, 100]	75.4±24.7<br>80 [40, 100]

Abbreviations: CA=Corrected age; ITT=Intention-to-treat; PP=Per protocol set

Supplementary Table 4. Early Child Behaviour Questionnaire, Short Form (ECBQ-SF), at 24 months CA, ITT population (N=23) and PP population (N=13)

**Score**	**ITT (N=23)**<br>**Mean±SD**<br>**Median [Range]**	**PP (N=13)**<br>**Mean±SD**<br>**Median [Range]**
**Surgency**	4.8±0.5<br>4.8 [3.7, 5.8]	4.8±0.7<br>4.7 [3.7, 5.8]
Impulsivity	5.1±0.7<br>5 [4, 6.8]	5.2±0.7<br>5 [4.3, 6.8]
Activity Level/Energy	4.4±0.7<br>4.1 [3.4, 5.9]	4.4±0.6<br>4.5 [3.4, 5.1]
High-intensity Pleasure	4.4±1.2<br>4.8 [2, 5.7]	4±1.3<br>4.5 [2, 5.7]
Sociability	5±1.2<br>5.8 [2.8, 6.3]	5.2±1.1<br>5.8 [3.3, 6.3]
Positive Anticipation	5.2±0.9<br>5.6 [3.4, 6.3]	5±1.0<br>5.2 [3.4, 6.0]
**Negative affect**	3.1±0.5<br>3.2 [2, 3.9]	3±0.6<br>3.1 [2, 3.9]
Discomfort	3.3±1.3<br>3 [1, 5.8]	2.8±1.1<br>2.6 [1, 4.7]
Fear	2.6±0.9<br>2.6 [1.1, 4.4]	2.4±1.1<br>2.8 [1.1, 4.4]
Motor Activation	2.3±1.1<br>1.8 [1, 5]	2.5±1.0<br>2.5 [1, 4.2]
Sadness	2.9±0.9<br>3 [1.5, 4.5]	2.8±0.7<br>3 [1.7, 3.8]
Perceptual Sensitivity	4.8±1.1<br>4.8 [2.8, 7]	4.5±1.0<br>4.6 [2.8, 5.7]
Shyness	3.5±1.2<br>3.3 [1.4, 5.4]	3.2±1.2<br>3.2 [1.4, 5.4]

Score	ITT (N=23)<br>Mean±SD<br>Median [Range]	PP (N=13)<br>Mean±SD<br>Median [Range]
Soothability	5.1±0.7<br>5 [3.4, 6.4]	5.1±0.8<br>5 [3.4, 6.4]
Frustration	2.9±0.9<br>3 [1.5, 4.8]	2.8±0.8<br>3 [1.5, 4.2]
**Effortful control**	5.2±0.6<br>5.3 [3.9, 6]	5.1±0.6<br>5.2 [3.9, 5.8]
Attentional Focusing	5.3±0.8<br>5.5 [3.5, 6.6]	5.2±0.9<br>5.3 [3.5, 6.5]
Attentional Shifting	5.1±0.8<br>5.1 [3.6, 6.8]	4.8±0.8<br>4.8 [3.6, 5.9]
Cuddliness	5.8±0.8<br>6 [3.8, 6.8]	5.9±0.5<br>6 [5.2, 6.8]
Low-intensity Pleasure	5.4±0.7<br>5.7 [3.8, 6.7]	5.3±0.9<br>5.3 [3.8, 6.7]
Inhibitory Control	4.3±0.9<br>4.5 [2, 5.7]	4.1±1.0<br>4 [2, 5.3]

Abbreviations: CA=Corrected age; ITT=Intention-to-treat; PP=Per protocol set

Supplementary Table 5. Hospitalization/Healthcare usage questionnaire at 24 months CA, ITT population (N=23) and PP population (N=13)

Measure	ITT (N=23) N (%)	PP (N=13) N (%)
Healthcare consultation	22 (95.6%)	12 (92.3%)
Type of practitioner		
Cardiologist	2 (8.7%)	0 (0%)
Endocrinologist	1 (4.4%)	1 (7.7%)
Gastroenterologist	1 (4.4%)	1 (7.7%)
Neurologist	2 (8.7%)	2 (15.4%)
Ophthalmologist	3 (13.0%)	3 (23.1%)
Physical therapist	17 (73.9%)	10 (76.9%)
Pneumologist	4 (17.4%)	2 (15.4%)
Pediatrician/ general practitioner	20 (87.0%)	12 (92.3%)
Risk care ambulance	2 (8.7%)	2 (15.4%)
Urologist	1 (4.4%)	1 (7.7%)
Number of visits		
0 - 5	14 (60.9%)	9 (69.2%)
6 - 10	12 (52.2%)	10 (76.9%)
11 - 15	3 (13.0%)	2 (15.4%)
16 - 20	4 (17.4%)	1 (7.7%)
21 - 25	1 (4.4%)	1 (7.7%)
Regularly	10 (43.5%)	4 (30.8%)
2 times per week	1 (4.4%)	1 (7.7%)
Type of visit		
Follow-up visit	5 (21.7%)	4 (30.8%)
Infection	1 (4.4%)	1 (7.7%)
Scheduled routine visit	21 (91.3%)	12 (92.3%)
Unscheduled + follow-up visits	1 (4.4%)	1 (7.7%)
Hospitalization	9 (39.1%)	7 (53.9%)
Duration of hospitalization, days [a]	4.2±2.4	5±2.1

Abbreviations: CA=Corrected age; ITT=Intention-to-treat; PP=Per protocol set

[a] Values are means±SD.

Supplementary Table 6. Feeding practices at 24 months CA, ITT population (N=23) and PP population (N=13)

**Measure**	**ITT (N=23)**	**PP (N=13)**
Ever breastfed	8 (34.8%)	3 (23.1%)
Age when breastfeeding stopped, months [a]	4 [1,12]	12 [1,12]
Ever consumed formula	23 (100%)	13 (100%)
Formula Type		
Preterm	13 (56.5%)	7 (53.9%)
Term	11 (47.8%)	7 (53.9%)
Specialty [b]	9 (39.1%)	4 (30.8%)
Age when complementary feeding introduced, months [a]	6 [4,12]	5.5 [4,12]
Consumption of other beverages		
Fortified cow's milk	1 (4.4%)	1 (7.7%)
Regular cow's milk	8 (34.8%)	4 (30.8%)
Growing up milk	6 (26.1%)	4 (30.8%)
Other type of milk	16 (69.6%)	10 (76.9%)

Abbreviations: CA=Corrected age; ITT=Intention-to-treat; PP=Per protocol set

[a] Values are medians [range].

[b] Specialty refers to formulas such as hypoallergenic or with partially hydrolyzed protein, etc.

REFERENCES

1. Bayley N, Infant S. Bayley scales of infant and toddler development–Third Edition: Technical manual. 2006.
2. Centers for Disease Control and Prevention. CDC's Developmental Milestones. https://www.cdc.gov/ncbddd/actearly/milestones/index.html. Published 2023. Accessed.
3. Putnam SP, Jacobs, J., Garstein, M.A., Rothbart, M.K.,. Development and assessment of short and very short forms of the early childhood behavior questionnaire. Paper presented at: International Conference on Infant Studies2010; Baltimore, MD.
